# Whole-Genome Sequencing of *Lactobacillus helveticus* D75 and D76 Confirms Safety and Probiotic Potential

**DOI:** 10.3390/microorganisms8030329

**Published:** 2020-02-26

**Authors:** Vyacheslav Toropov, Elena Demyanova, Olga Shalaeva, Stanislav Sitkin, Timur Vakhitov

**Affiliations:** 1 Department of Microbiology, State Research Institute of Highly Pure Biopreparations, 197110 St. Petersburg, Russia; v.a.toropov@hpb.spb.ru (V.T.); e.v.demyanova@hpb.spb.ru (E.D.); o.n.shalaeva@hpb.spb.ru (O.S.); drsitkin@gmail.com (S.S.); 2 Department of Internal Diseases, Gastroenterology and Dietetics, North-Western State Medical University named after I.I. Mechnikov, 195067 St. Petersburg, Russia

**Keywords:** *Lactobacillus helveticus*, probiotics, whole genome sequencing, PacBio, probiotic genes, bacteriocins, gene expression

## Abstract

Whole-genome DNA sequencing of *Lactobacillus* D75 and D76 strains (Vitaflor, Russia) was determined using the PacBio RS II platform, which was followed by de novo assembly with SMRT Portal 2.3.0. The average nucleotide identity (ANI) test showed that both strains belong to the *Lactobacillus helveticus*, but not to the *L. acidophilus*, as previously assumed. In addition, 31 exopolysaccharide (EPS) production genes (nine of which form a single genetic cluster), 13 adhesion genes, 38 milk protein and 11 milk sugar utilization genes, 13 genes for and against specific antagonistic activity, eight antibiotic resistance genes, and also three CRISPR blocks and eight Cas I-B system genes were identified in the genomes of both strains. The expression of bacteriocin helveticin J genes was confirmed. In fact, the presence of identified genes suggests that *L. helveticus* D75 and D76 are able to form biofilms on the outer mucin layer, inhibit the growth of pathogens and pathobionts, utilize milk substrates with the formation of digestible milk sugars and bioactive peptides, resist bacteriophages, show some genome-determined resistance to antibiotics, and stimulate the host’s immune system. Pathogenicity genes have not been identified. The study results confirm the safety and high probiotic potential of the strains.

## 1. Introduction

*Lactobacillus acidophilus* D75 and D76 have been used as components of the Vitaflor probiotic (St. Petersburg, Russian Federation) since 1997. The combination of two closely related strains in one probiotic product is due to marked symbiotic relationships between them, which leads to synergism and certain properties that underpin their probiotic activity, including increasing the titre of viable bacteria during cultivation, enhancing resistance to environmental stress, and stimulating antagonism against pathogens [1]. Previous clinical studies have shown that *L. acidophilus* D75 and D76 possess unique probiotic activity. These strains demonstrated efficacy for treating chronic nasopharyngeal disease [2], eradicating *Helicobacter pylori* infection in children with gastroduodenal lesions [3], treating of mucosa-associated lymphoid tissue (MALT) lymphoma (when antibiotic treatment proved ineffective) [4,5], treating paediatric patients with vulvovaginitis [6], treating cancer patients with complications due to chemotherapy and radiation therapy [4], treating *Klebsiella* spp. infection [7], and treating inflammatory bowel diseases [8]. In patients with ulcerative colitis receiving corticosteroids, *L. acidophilus* D75 and D76 can suppress the excessive growth of *Candida* spp. in the colon [8].

*L. acidophilus* D75 and D76 can increase the effectiveness of influenza vaccines and pneumococcal vaccines. The incidence of pneumonia, acute bronchitis, and acute respiratory infections among people vaccinated with both vaccines and receiving *L. helveticus* D75 and D76 simultaneously was 2.9-fold and between 2.5-fold and 4.1-fold lower than those not receiving probiotics. In addition, the severity of pneumonia in vaccinated individuals receiving probiotics was significantly reduced [9].

For these reasons, the genetic characteristics of these strains were interested in studying primarily those associated with their probiotic activity. Given the molecular genetic data acquired in recent decades, it seems appropriate to clarify the systematics of bacterial strains using advanced phylogenetic analysis, specifically by calculating and comparing average nucleotide identity (ANI) between the genomes of novel and previously annotated strains. Since these strains are widely used as dietary supplements in Russia, it may also be useful to ensure that they lack the genetic determinants of virulence as well as genes encoding the synthesis of toxic metabolites and antibiotic resistance genes.

## 2. Materials and Methods

### 2.1. Bacterial Strains

*L. acidophilus* D75 and D76 served as test strains in our research. These strains were acquired by the Museum of Bacterial Cultures of the State Research Institute of Highly Pure Biopreparations of the FMBA of the Russian Federation. Both D75 and D76 strains were isolated from gastrointestinal tract of a healthy child in St. Petersburg in 1991. Strains D75 and D76 have been deposited in the Departmental Collection of Useful Microorganisms of Agricultural Importance, registered in the WDCM database under international catalogue numbers RCAM04483 (D75) and RCAM04484 (D76).

### 2.2. Whole-Genome Sequencing and the Phylogenetic Analysis of Lactobacillus Strains D75 and D76

*Lactobacillus* strains D75 and D76 were cultured on Man Rogosa Sharpe (MRS-1) medium 37 °C for 6 h [10]. Isolation of DNA was carried out according to a method described previously [11].

Both genomic DNAs were sequenced using Single-Molecule Real-Time sequencing technology (SMRT) on a PacBio RS II instrument (Macrogen Inc., Seoul, South Korea Seoul Capital Area, Republic of Korea). Subsequent genomic assembly was performed using the SMRT Portal software package (version 2.3.0, Pacific Biosciences, Menlo Park, CA, USA) [12].

The Prokaryotic Genome Annotation Pipeline (PGAP) algorithm (NCBI, Bethesda, MD, USA) was employed *de novo* genome annotation [13], along with Artemis (version 16, Wellcome Sanger Institute, Hinxton, Cambridgeshire, UK) [14], Mauve (version 2.4.0, the Darling lab, New South Wales Institute of Technology, Sydney, New South Wales, Australia) [15], ISfinder database [16], Basic Local Alignment Tool (BLAST, NCBI, Bethesda, MD, USA) [17], Pfam [18], KEGG [19], and UniProt [20] databases.

Phylogenetic identification was performed via Average Nucleotide Identity (ANI) analysis of DNA from strains D75, D76, and reference organisms [21,22] using JSpecies (version 1.0, developed by Richter M. and Rosselló-Mora R. for the Marine Microbiology Group, IMEDEA, Esporles, Illes Balears, Spain) and OrthoANI (version 2017-06-14, Seoul National University, Gwanak, Seoul, Republic of Korea) [23,24]. Genomic analysis was also carried with the BLAST algorithm. Fully sequenced and annotated genomes (*Lactobacillus acidophilus* strains NCFM, La-14, ATCC 4356, 4796, CFH, FSI4, WG-LB-IV, and *Lactobacillus helveticus* strains DPC4571, R0052, CNRZ32, H9, H10, and CAUH18) were obtained from the NCBI database as comparison genomes.

### 2.3. Identification of Genetic Determinants Responsible for the Probiotic Properties of L. helveticus D75 and D76

Artemis and Mauve programs were used for a manual search of target genes in the *L. acidophilus* D75 and D76 genomes. InterPro [25], KEGG, NCBI [26], and CRISPRfinder [27] were used for functional analysis of the identified genes. Analysis of protein domains and their functions was performed using the Pfam database.

### 2.4. Isolation and Purification of Total RNA from L. helveticus D75 and D76

Cells were precipitated by centrifugation, washed twice in 0.85% saline, and 20 mg of cell precipitate was suspended in 50 µL of 10 mM TRIS-HCl buffer (pH 8.0). A 10-µL sample of 10 mg/mL lysozyme (Sigma-Aldrich, St. Louis, MO, USA) was added and incubated at 37 °C for 15 min. After incubation, 1 mL of TRIzol Reagent (Sigma-Aldrich, St. Louis, MO, USA) was added to the mixture and incubated at room temperature for 20 min. A 200-µL sample of chloroform (Sigma-Aldrich, St. Louis, MO, USA) was added to the resulting mixture, stirred, and incubated for 10 min at room temperature. The mixture was centrifuged for 5 min at 13,000 *g* with a 54,150 instrument (Eppendorf, Hamburg, Germany). An equal volume of isopropanol (Sigma-Aldrich, St. Louis, MO, USA) was added to 600 µL under an aqueous phase, stirred, and incubated at 4 °C for 10 min. After incubation, the mixture was centrifuged at 13,000 *g* for 5 min.

The supernatant was removed, the RNA pellet was washed with 76% ethanol solution, dried for 5 min at 16 °C, 50 μL of sterile deionised water was added, and the resulting RNA preparation was stored at −70 °C until use.

### 2.5. Preparing cDNA from Total RNA of L. helveticus D75 and D76

A total of 8 μL of total RNA preparation, 1 μL of 10 DNAse buffer (Thermo Fisher Scientific, Waltham, MA, USA), and 1 μL of DNAse (Thermo Fisher Scientific, Waltham, MA, USA) were successively added and incubated for 15 min at room temperature. The action of DNAse was stopped by the addition of 50 mM ethylenediaminetetraacetic acid (EDTA) solution (Thermo Fisher Scientific, Waltham, MA, USA) to a 5 mM final concentration and incubation at 70 °C for 10 min. The resulting pure total RNA solution was used in the reverse transcription reaction. Specifically, 5 µL of total RNA (0.6 μg/μL) and 0.5 µL of random primers (Thermo Fisher Scientific, Waltham, MA, USA) were introduced to 10 µL of sterile water, incubated at 70 °C for 2 min, and 4 µL of a ten-fold reverse transcription buffer (Thermo Fisher Scientific, Waltham, MA, USA), 2 µL of dNTPs (Thermo Fisher Scientific, Waltham, MA, USA), and 1 µL of MMuLV reverse transcriptase (Sintol, Moscow, Russia) were added. The volume of the mixture was adjusted to 20 µL with sterile deionised water, the mixture was incubated for 5 min at 25 °C, and the reverse transcription reaction was performed for 45 min at 37 °C. The reaction was stopped by heating for 10 min at 70 °C.

### 2.6. Real-Time PCR

To analyse gene expression, a commercial real-time PCR kit (Syntol, Moscow, Russia) was employed along with an iQ5 amplifier instrument (Bio-Rad, Hercules, CA, USA). The reaction contained 1 µL cDNA solution (50 ng/µL), 1 µL dNTPs (Syntol, Moscow, Russia), 2.5 µL tenfold PCR buffer (Syntol, Moscow, Russia), 2.5 µL MgCl_2_ (Syntol, Moscow, Russia), 0.5 µL direct and reverse primers (100 pmol), 0.3 µL SynTaq DNA polymerase (Thermo Fisher Scientific, Waltham, MA, USA), and sterile deionised water (Syntol, Moscow, Russia) to a final volume of 25 µL.

Amplification involved three stages. The first stage included preliminary melting of double-stranded DNA and activation of the polymerase at 95 °C for 5 min. The second stage involved 40 cycles of amplification comprising melting of double-stranded DNA molecules at 95 °C (15 s), primers annealing at 53.5 °C for 15 s, amplicon elongation at 72 °C for 15 s, and reading of the SYBRGreen fluorescence signal at 72 °C for 30 s. The final stage involved constructing melting curves for products in accordance with the recommendations of the iQ5 amplified manufacturer. Primer sequences are listed in Table 1.

## 3. Results

### 3.1. Whole-Genome Sequencing and Phylogenetic Analysis of L. helveticus D75 and D76

**Figure 1:**
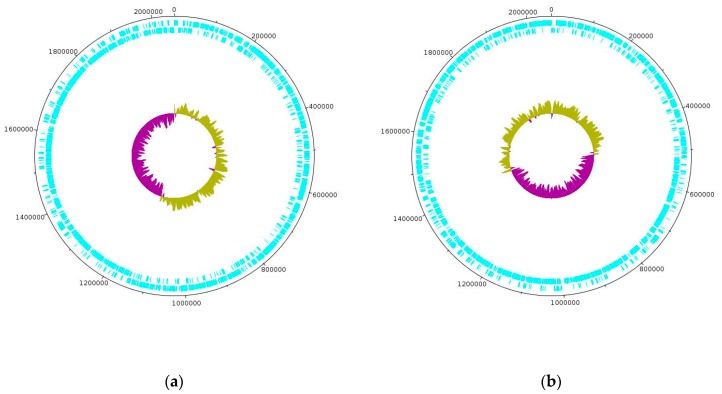
Genetic maps of circular closed genomes strains of *L. helveticus* D75 (**a**) and *L. helveticus* D76 (**b**). The coding nucleotide sequences for the direct (outer circle) and reverse (inner circle) strands of genomic DNA are coloured blue in the outer ring of the genetic map, while the inner ring shows the relatively high (olive) and slightly lower (dark magenta) GC ratio for D75 (**a**) and D76 (**b**).

Whole-genome sequencing on the PacBio platform yielded the complete genome sequences for strains D75 and D76 spanning 2,053,066 base pairs (bp) and 2,058,319 bp, respectively. The D75 and D76 sequences have been uploaded to the GenBank database for newly sequenced and annotated genomes under accession codes CP020029 and CP16827, respectively. The GC content of both genomes is 37%. The D75 and D76 genomes contain 2092 and 2068 coding sequences (CDS), respectively, as well as 317 and 265 pseudogenes [11]. Using the ISfinder program, IS elements from 67 families with an E-value from 0.0 to 0.0002 were identified in both genomes. It is interesting that the bacterial genomes are made up of two halves including one of which is characterized by an overestimated and an underestimated amount of GC pairs. In this case, when the number of GC pairs is overestimated, the direct genome chain is mainly used for coding while the reverse chain is used when the number is underestimated. Figure 1 shows genetic maps of the circular-closed genomes of strains D75 and D76.

According to the ANI calculation, the genomes of strains D75 and D76 share 99.22% sequence identity (with 76.3% overlapping nucleotide sequences) with other genomes of *L. helveticus* species. BLAST alignment of the two genomes revealed exceptionally high sequence similarity of 99.9% (with 99% overlapping nucleotide sequences). The main differences between the genomes is the presence of four additional copies of genes related to IS elements in strain D76, 28 single nucleotide substitutions at various positions, four deletions of sections of IS elements up to 1400 bp long, and one 307 bp deletion in the genome of strain D76 within an S8 family peptidase gene.

Initially, based on phenotypic traits, strains D75 and D76 were assigned to the species *L. acidophilus* [1]. However, after whole-genome sequencing and ANI analysis, strains D75 and D76 were found to be 99.18% (with 90.65% overlapping nucleotide sequences) and 97.73% (with 80.45% overlapping nucleotide sequences) identical to the genomes of strains *L. helveticus* DPC4571 and R0052, respectively. Furthermore, the genomes of strains D75 and D76 share only 80.54% to 80.62% sequence similarity (over 70% overlapping nucleotide sequences) with *L. acidophilus* (strains NCFM, La-14, ATCC 4356, 4796, CFH, FSI4, WG-LB-IV) below the threshold value of 95% required to confirm their belonging to these previously characterized bacterial species [28].

In addition, we performed a general analysis of the genomes of *L. helveticus* and *L. acidophilus* species available in the NCBI database. The results showed that the presence of a large number of pseudogenes in strains D75 and D76 (~200–300) is more typical of *L. helveticus* than *L. acidophilus* (~20–30). Similarly, the GC content also corresponds more closely to that of *L. helveticus* (~37%) than *L. acidophilus* (~34%). These results confirm the correctness of the assignment of strains D75 and D76 to the species *L. helveticus* based on the ANI method.

### 3.2. L. helveticus D75 and D76 Genes Involved in the Synthesis of Exopolysaccharides

**Figure 2:**
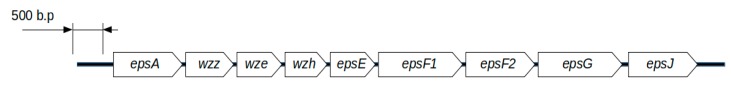
The genetic cluster related to the synthesis of heteropolymer exopolysaccharides in *L. helveticus* D75 and D76. The location of the start of the genetic cluster in *L. helveticus* D75 is 284699.293492 bp, while, in *L. helveticus* D76, it is 1719263.1728034 bp.

During genome annotation, 31 genes were found to be involved in the synthesis of exopolysaccharides in *L. helveticus* D75 and D76, among which nine form a single genetic cluster (Figure 2).

The *epsA* transcription regulator gene encodes the LytR_CpsA_psr protein domain. According to information in the InterPro, KEGG, and Pfam databases, transcription regulator proteins with the LytR_CpsA_psr domain are involved in the regulation of various cellular processes, including exopolysaccharide synthesis, exoprotein expression, and bacterial quorum sensing [29]. In the case of *L. helveticus* D75 and D76, the *epsA* gene encodes a putative regulatory protein involved in exopolysaccharide biosynthesis since it is part of a single genetic cluster along with genes related to the synthesis of exopolysaccharides, and is located in accordance with the conserved structure of exopolysaccharide clusters in lactic acid bacteria (LAB) [30]. The size of the protein product is 353 amino acids.

The *wzz* gene encodes a protein containing a polysaccharide chain length determinant N-terminal domain. According to the InterPro database, this protein domain is involved in regulating the length of the synthesised polysaccharide chain, which promotes the formation of longer molecules. A number of articles indicate that the protein encoded by the *wzz* gene is a polysaccharide co-polymerase [31,32]. The size of the protein product is 295 amino acids.

The *wze* gene encodes a P-loop protein containing a nucleoside triphosphate hydrolase and an ATPase domain associated with diverse cellular activities (AAA domain) [33,34]. P-loop NTP hydrolases catalyse energy-consuming conformational changes in macromolecules. Most often, ATP or GTP act as the energy substrate for P-loop NTPases. According to the InterPro database, the protein encoded by the *wze* gene is involved in the synthesis of exopolysaccharides. The size of the protein product is 264 amino acids.

The *wzh* gene encoding the manganese-dependent protein-tyrosine phosphatase is presumably responsible for regulating the synthesis of exopolysaccharides [35,36]. According to the InterPro database, the protein belongs to the polymerase and histidinol phosphatase (PHP) transferase family and consists of 256 amino acids.

The *epsE* gene encoding the priming-glycisyltransferase protein is a bacterial sugar transferase. Presumably, this protein is responsible for the formation of normal glycosidic bonds in the exopolysaccharide capsule. It catalyses the initial stage of the synthesis of exopolysaccharide molecules and is necessary for the transfer of the first sugar molecule from sugar 1-phosphate to a phosphorylated carrier lipid [30]. The size of the protein product is 216 amino acids. This protein is necessary for the proper functioning of any exopolysaccharide’s gene cluster. In the genome of *L. helveticus* D76, the *epsE* gene is a pseudogene due to the presence of an internal stop codon. In the genome of *L. helveticus* D75, the gene is active. The most important phenotypic difference between strains D75 and D76 is the difference in the production of exopolysaccharide mucus. Perhaps the presence of a stop codon in the *epsE* gene in strain D76 is the cause of this difference.

The *epsF1* and *epsF2* genes encode two glycosyltransferase family 1 enzymes, which are 371 (*epsF1*) and 360 (*epsF2*) amino acids, respectively. Each protein includes two protein domains: a glycosyltransferase family 1 domain and a glycosyltransferase subfamily 4-like, N-terminal domain.

The first domain is responsible for the formation of glycosidic bonds by transferring sugar moieties of donor molecules to specific acceptor molecules. Proteins possessing this domain transfer UDP, ADP, GDP, or CMP-jointed saccharides to various acceptor substrates, such as glycogen, fructose-6-phosphate, and lipopolysaccharides. In bacteria, proteins with this domain can be involved in processes such as the biosynthesis of exopolysaccharides, the biosynthesis of lipopolysaccharides, and the production of slime polysaccharide colanic acid.

The second domain is an integral component of the cell wall. According to the UniProt database, this domain has two types of activity: phospho-N-acetylmuramoyl-pentapeptide-transferase activity (GO: 0008963) and UDP-N-acetylglucosamine-undecaprenyl-phosphate N-acetylglucosaminephosphotransferase activity (GO: 0036380). The first activity is necessary for the synthesis of peptidoglycan of the bacterial cell wall, and the second activity is needed for the biosynthesis of various polymers of the cell envelope. Thus, EpsF1 and EpsF2 proteins may be involved in both the formation of extracellular polysaccharides and the biosynthesis of the bacterial cell wall.

The hypothetical *epsG* gene encodes an exopolysaccharide biosynthesis protein containing an EpsG family protein domain belonging to the glycosyltransferase domain class, and belonging to the GT-C membrane proteins. The gene shares a low homology with similar genes in the KEGG database. The size of the protein product is 372 amino acids.

The *epsJ* gene encodes an enzyme with potential nucleotide-diphospho-sugar transferase activity, based on amino acid sequence alignment with reference sequences in the InterPro database. The protein product of the gene does not include domains synonymous with those known in the Pfam database. However, in *L. helveticus* D75 and D76, this gene is part of a single genetic cluster for the synthesis of exopolysaccharides. Thus, it can be assumed that it is involved in the synthesis of exopolysaccharides in *L. helveticus* D75 and D76. The size of the protein product is 338 amino acids.

During the analysis, a gene potentially encoding a flippase transporter (*wzx*) was identified in the genomes of *L. helveticus* D75 and D76. The gene is not part of the genetic cluster for the synthesis of exopolysaccharides, but plays a key role in their synthesis along the WZX-WZY-dependent pathway. Flippase transporters transfer polysaccharide precursor molecules from the cytoplasmic space to the external pre-membrane region [37,38,39]. To synthesise exopolysaccharides via the WZX-WZY-dependent pathway, the *wzy* gene is also required, which encodes a polysaccharide polymerase protein that, together with the polysaccharide co-polymerase protein (encoded by the *wzz* gene), polymerises the growing chain of the exopolysaccharide. The gene itself and the protein encoded by it can differ significantly in nucleotide and amino acid sequence among various exopolysaccharide-producing bacterial species. The machinery for the synthesis of exopolysaccharides along the Wzx-Wzy pathway is present in the vast majority of Gram-positive exopolysaccharide-producing bacteria [40].

A gene similar to the *wzy* genes of other exopolysaccharide-producing bacteria present in reference databases (Pfam, KEGG, InterPro, NCBI) as well as among the genus *Lactobacillus* [40], which was not found in the genomes of *L. helveticus* D75 and D76. However, in vitro experiments on *L. helveticus* D75 and D76 confirmed the synthesis of exopolysaccharides. This suggests that the genomes of these bacteria, nevertheless, contain an analogue of the highly variable *wzy* gene.

In addition to the nine-gene cluster, a further 22 genes distributed throughout the genome are responsible for the synthesis of the cell wall and exopolysaccharides (Supplementary, Table S1).

### 3.3. Genes Encoding Surface Proteins

In addition to complex supramolecular compounds, such as pili and fimbriae, there are single proteins that facilitate the adhesion of bacteria to the surface structures of various host cells [41].

*L. helveticus* D75 and D76 have a set of genes encoding single protein products that allow them to bind to fibrinogen and mucin due to the presence of the appropriate domains. Adhesive proteins include a mucin-binding protein (encoded by *mucBP*), fibronectin A (encoded by *fpbA*), and an S-layer protein (encoded by *slpA*).

Fibronectin A, which is encoded by the *fpbA* gene, consists of two domains that include a type I fibronectin-binding N-terminal domain and an NFACT protein RNA-binding domain. The first domain is located on the surface of the bacterial cell membrane and is a dimerised glycoprotein. The second domain is a domain of unknown function (DUF, PF05670 in the Pfam database). The protein product of the *fpbA* gene is believed to bind to the fibrillar network on the cell surface and on the extracellular matrix of mammalian cells, including gastrointestinal cells. The size of the protein product is 564 amino acids.

The *fpbIII* gene, containing a fibronectin type III domain, is located on the surface of the bacterial cell wall, and is involved in adhesion to the surface fibrinogen of mammalian cells. The size of the protein product is 464 amino acids.

The *mucBP* gene encoding a mucin-binding protein contains a domain that binds to mucin. It is assumed that this domain allows bacterial cells to adhere to the mucin layer of the gastrointestinal tract [18,42]. The genomes of *L. helveticus* D75 and D76 contain six variants of this gene ranging from 55 to 228 amino acids in length.

The *srtA* gene contains a sortase protein domain. Sortase proteins attach substrate proteins to the cell wall of bacteria during the adhesion process [43]. The size of the protein product is 229 amino acids.

In addition, *slpA* genes encoding S-layer proteins were identified in the genomes of *L. helveticus* D75 and D76. According to the literature, S-layer proteins enhance the immunostimulatory activity and adhesion of bacteria to epithelial cells [43,44]. The genomes of *L. helveticus* D75 and D76 contain four variants of *slpA* genes ranging in length from 166 to 454 amino acids. The main genetic structures mediating adhesive properties and their positions in the genomes of *L. helveticus* D75 and D76 are presented in Supplementary, Table S2.

### 3.4. Genetic Structures Involved in Casein Utilisation

*L. helveticus* D75 and D76 possess 38 genes encoding various proteases and peptide transport systems. Among the identified proteases, extracellular proteinase lactocepin H3 (encoded by *prtH3*) and extracellular proteinase lactocepin H (encoded by *prtH*) interact with casein directly and break it into digestible oligopeptides and tripeptides. The protein product of the *prtH3* gene contains four domains (an S8 peptidase domain, an Fn3-like domain, and two SlpA domains) located from the N-terminus to the C-terminus [18,42]. The S8 peptidase domain is a subtilase (serine protease) possessing endopeptidase or, in some cases, exopeptidase activity. The Fn3-like domain is a fibronectin type III protein with the ability to adhere to a protein substrate. The SlpA domains are surface layer protein A molecules. Lactocepin H3 protein binds to the surface of the bacterial cell membrane using the SlpA domain and is able to effectively interact with milk casein in the extracellular space. The size of the protein product is 1596 amino acids.

The *prtH* gene encodes a protein consisting of an I9 peptidase inhibitor domain, an S8 peptidase domain, a protease-associated (PA) domain, an Fn3-like domain, and an SlpA domain (from the N- to the C-terminus). The I9 peptidase inhibitor domain blocks the catalytic centre of the protein, and inhibits its peptidase activity. The PA domain forms a lid-like structure that covers the active site in active proteases [18,42]. The presence of this domain is required to regulate protease activity and its activation only on the outer surface of the cell when in contact with specific substrates [45]. The other lactocepin H protein domains share similar functions. The size of the protein product is 1761 amino acids.

In addition to the proteins encoded by *prtH3* and *prtH*, endopeptidase O2 (*pepO2*), and endopeptidase O (*pepO*) play a significant role in the utilisation of casein and milk oligopeptides. Endopeptidases consist of two domains: M13 N and M13 peptidases. Both domains possess peptidase enzymatic activity and are involved in milk protein fermentation [18,42]. The size of the protein products is 648 and 647 amino acids, respectively.

Proteolytic enzymes and systems that transport oligopeptides and amino acids into the intracellular space and their positions in the genomes of *L. helveticus* D75 and D76 are presented in Supplementary, Table S3.

### 3.5. Genetic Structures Involved in the Utilisation of Milk Sugars

An important characteristic of LAB is their ability to metabolise lactose from milk [46]. Genetic analysis of *L. helveticus* D75 and D76 revealed the presence of lactose permease (*lacS*), transcriptional regulator LacI family (*lacR*), a large beta-galactosidase subunit (*lacL*), and small beta-galactosidase subunit (*lacM*) genes.

Lactose permease (*lacS*) is a membrane transport protein responsible for the transfer of lactose molecules into the cell. It consists of two protein domains: a major facilitator superfamily (MFS)/sugar transport protein and a phosphoenolpyruvate-dependent sugar phosphotransferase system (EIIA 1) domain. The size of the protein product is 638 amino acids. The transcriptional regulator (*lacR*) has two domains: a bacterial regulatory protein (lacI family) and a periplasmic binding protein-like domain. The size of the protein encoded by the *lacR* gene is 335 amino acids. The *lacL* and *lacM* genes are responsible for the synthesis of proteins involved in the formation of the β-galactosidase complex that hydrolyses β-galactosides, including lactose. The proteins encoded by the *lacL* and *lacM* genes are 628 and 318 amino acids, respectively. Supplementary, Table S4 lists the genes involved in the process of lactose utilisation in *L. helveticus* D75 and D76, along with their positions in the genomes.

In the genomes of *L. helveticus* D75 and D76, genes were identified that encode proteins of the Leloir metabolic pathway characteristic of lactobacilli [46]. Due to the presence of galactose mutarotase (*galM*), galactose-1-phosphate uridylyltransferase (*galT*), galactokinase (*galK*), and UDP-galactose-4-epimerase (*galE*) genes, these strains can convert D-galactose to UDP-glucose. Supplementary, Table S5 lists the Leloir pathway genes and their positions in the genomes of *L. helveticus* D75 and D76.

### 3.6. Genetic Structures Encoding CRISPR Sequences and Cas Proteins

In the genomes of *L. helveticus* D75 and D76, genes related to the CRISPR-Cas type I-B system were discovered. It is believed that CRISPR-Cas systems act as adaptive immunity in bacterial cells to counter foreign pathogens such as bacteriophages. In addition, CRISPR-Cas systems are involved in the regulation of gene activity, DNA repair, genome reorganisation, and the translation of a bacterial cell into an inactive (anabiosis) state [47,48].

The nucleotide sequences of CRISPR spacers play a key role in the recognition of foreign genetic material. Upon contacting marker sites, they contribute to the formation of CRISPR-Cas complexes and subsequent degradation of foreign genetic material. In the genomes of *L. helveticus* D75 and D76, three blocks of CRISPR arrays were detected. In total, they contain 33 spacer and direct repeating sequences. The CRISPR1 block contains three spacers and four short, direct repeating sequences. The CRISPR2 block has nine spacers, seven short, direct repeating sequences, and one non-repeating sequence. The CRISPR3 array contains 21 spacers and 21 short direct repeating sequences. Among the short direct repeating sequences, there is one sequence with an A to C nucleotide substitution at position 740 in the CRISPR3 array (position 1,492,878 in the genome of *L. helveticus* D75 and 871,612 in the genome of *L. helveticus* D76). The Cas system is represented by the following genes: *cas1* (encoding a 329 amino acid protein), *cas2* (encoding a 93 amino acid protein), *cas3* (encoding a 809 amino acid protein), *cas4* (encoding a 163 amino acid protein), *cas5* (encoding a 237 amino acid protein), *cas6* (encoding a 251 amino acid protein), *cas7* (encoding a 300 amino acid protein), and *cas8b1* (encoding a 585 amino acid protein). All Cas and CRISPR genes are listed in Supplementary, Table S6.

### 3.7. The Genetic Mechanisms Responsible for Specific Antibacterial Antagonism

**Figure 3:**
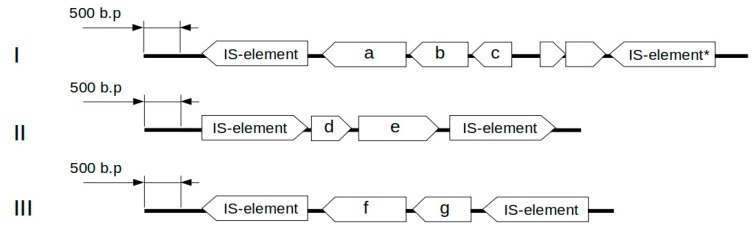
Clusters of genes related to the synthesis of the bacteriocin helveticin J in *L. helveticus* D75 and D76. (**I**) The genetic cluster consisting of *slpA1* (a), *helJ1* (b), and *helJ2* (c). (**II**) The genetic cluster consisting of *helJ3* (d) and *slpA2* (e). (**III**) The genetic cluster consisting of *slpA4* (f) and *helJ5* (g). Asterisks (*) present pseudogenes.

The genomes of *L. helveticus* D75 and D76 contain 13 genes involved in the formation of specific antagonism, including those encoding the bacteriocin helveticin J (Supplementary, Table S7). Three genetic clusters are presumably responsible for the synthesis of helveticin J, and a separate gene is related to the synthesis of prebacteriocin helveticin J. The genetic clusters include two types of open reading frames (ORFs) including a prebacteriocin helveticin J related gene and an *slpA* gene encoding a surface protein that is presumably involved in the synthesis of bacteriocin. Figure 3 shows the structures of the identified gene clusters.

Alignment using the BLAST program showed that the *slpA2* and *slpA4* encoding surface proteins share 86% nucleotide sequence identity and 88% amino acid sequence identity. By contrast, *slpA1* shares <30% amino acid sequence identity with the proteins encoded by *slpA2* and *slpA4*. The protein encoded by *slpA3* shares less than 20% identity with those encoded by *slpA1*, *slpA2*, and *slpA4*.

The results of nucleotide and amino acid sequence alignment of the *helJ1*-*helJ5* genes (BLAST, NCBI) with standard parameters are provided in Table 2 and Table 3. Only the *helJ3* and *helJ5* genes share high nucleotide sequence identity (77%). The *helJ4* gene in *L. helveticus* D75 and D76 shares high homology with similar genes in *L. helveticus* R0052 and DPC4571 [49]. The amino acid sequences encoded by *helJ1*, *helJ2*, *helJ3*, *helJ4*, and *helJ5* share moderate identity (Table 3) [50].

When *L. helveticus* D75 and D76 were routinely cultivated in MRS-1 medium at 37 °C without shaking, the expression level of the *helJ4* gene was 0.38 units relative to subunit B of the gyrase (*gyrB*) housekeeping gene. The *helJ4* gene was chosen due to its high homology with genes in the genomes of the sequenced and annotated strains *L. helveticus* DPC4571 and *L. helveticus* R0052 [51,52]. We suggest that this gene is inducible, and the low level of expression is most likely associated with the absence of the inducer in the culture medium. The characteristics of the primers used to amplify regions of the *helJ4* and *gyrB* genes are shown in Table 1.

### 3.8. Resistance to Bile

In the genomes of *L. helveticus* D75 and D76, two bile salt resistance genes were identified. The *bshA* gene encoding a bile salt hydrolase is located in the genomes of *L. helveticus* D75 and D76 at positions 854814.855791 bp and 232441.233418 bp, respectively. This gene has an internal termination codon and contains an CBAH protein domain and a linear amide C-N hydrolases domain belonging to the choloylglycine hydrolase family. The length of the *bshA* gene is 978 nucleotides.

The second gene acting on bile salts (*bshB*) is a GNAT family acetyltransferase and is located in the genomes of *L. helveticus* D75 and D76 at positions 1036556.1037308 bp and 414151.414903 bp, respectively. The *bshB* gene contains an CBAH protein domain as well as DUF4821 (a domain of unknown function) and Acetyltransf_1 domains. The protein product of the *bshB* gene is 250 amino acids in length.

### 3.9. Genes Underpinning Moderate Antibiotic and Xenobiotic Resistance in L. helveticus D75 and D76

The genomes of *L. helveticus* D75 and D76 possess eight genes associated with moderate antibiotic resistance and resistance against xenobiotics. All these genes are located on genomic DNA rather than on transposons or other mobile genetic elements. Antibiotic resistance-related genes in *L. helveticus* D75 and D76 include a penicillin-hydrolysing class A beta-lactamase BlaZ family protein (*blaZ*), a penicillin-binding protein (*pbpX*), a bifunctional lysylphosphatidylglycerol flippase/synthetase (*mprF*), and a 4-nitrophenylphosphatase (*had*). Supplementary Table S8 lists antibiotic resistance genes and their locations in the bacterial genomes.

The presence of the *blaZ* gene endows *L. helveticus* D75 and D76 with resistance to antibiotics such as penicillins, cephalosporins, monobactams, and carbapenems, which are more resistant to β-lactamases. Both strains additionally possess two different *pbpX* genes that likely provide additional penicillin resistance to other β-lactam antibiotics. *L. helveticus* D75 and D76 may have some resistance to teicoplanin, which is a glycopeptide lantibiotic active against Gram-positive bacteria.

*L. helveticus* D75 and D76 have a bifunctional lysylphosphatidylglycerol flippase/synthetase gene in their genome. This enzyme modifies the negative charge of membrane phosphatidylglycerol with a positively charged L-lysine, which leads to the repulsion of foreign cationic peptides, host immune system peptides, bacteriocins, defensins, and some other molecules.

### 3.10. Analysis of Pathogenicity Islands and Pathogenicity-Related Genes

Genomic analysis of *L. helveticus* D75 and D76 did not reveal pathogenicity islands or pathogenicity-related genes. This may indicate the safety of the strains for use as probiotics in humans.

A hemolysin III-like gene was identified in the genomes of *L. helveticus* D75 and D76. Identical genes are found in bacteria with proven probiotic functions that are known to be safe, such as *L. helveticus* KLDS1.8701, *L. helveticus* CNRZ32, *L. helveticus* DPC4571, *L. helveticus* H10, *L. gallinarum*, *L. helveticus* R0052, *L. crispatus*, and others (NCBI, KEGG). The position of the gene in the genome of *L. helveticus* D75 is 950,668.951,360 bp, and, in the genome of *L. helveticus* D76, it is 328,295.328,987 bp. None of the *L. helveticus* D75 and D76 have exhibited hemolytic activity [1].

## 4. Discussion

*L. helveticus* is very difficult to distinguish from *L. acidophilus* by phenotypic, biochemical, or morphological characteristics, and, regarding genetic classification, the variable regions of the 16S rRNA sequences of these species are 98.4% identical [51,52]. Thus, there is a need for fundamental genomic approaches for accurate species identification of new strains via sequencing, genomic, and bioinformatics analyses.

Our sequencing results confirmed that strains D75 and D76 are closely-related organisms (99.9% identity between nucleotide sequences in the genome). However, subtle differences between the strains were revealed. These include the presence of four additional copies of IS element genes in strain D76, 28 single nucleotide substitutions at various positions in the genomes of both strains, four deletions of sections of IS elements with lengths up to 1400 bp, and a 307 bp deletion of a locus in S8 family peptidase in strain D76.

As noted in Section 3.1, differences in DNA coding sequences (CDS) and pseudogenes were detected in strains D75 and D76. However, most of these discrepancies were caused by technical issues, which include incorrect automatic annotation in the Prokaryote Genome Annotation Pipeline (PGAP). Subsequent manual verification revealed that annotation errors were associated with an erroneous ORF offset, or with erroneous detection of stop codons. In some cases, PGAP did not annotate ORFs, which wrongly characterise them as non-coding regions of the genome.

**Figure 4:**
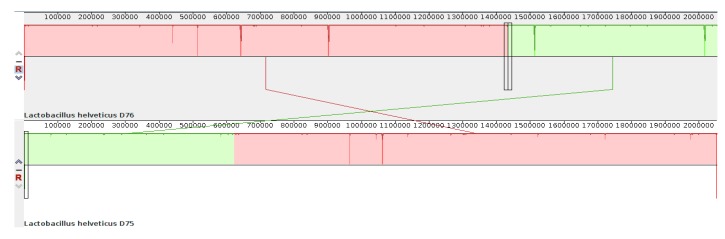
Location of genomic blocks in the genomes of *L. helveticus* D75 and D76 aligned with each other.

The numbering of bases in the genomes of strains D75 and D76 is also different. This results from the initial incorrect determination of the site of origin of replication in the *L. helveticus* D76 genome during automatic annotation in the PGAP pipeline (NCBI database). Figure 4 shows the location of the genomic blocks of strains D75 and D76 relative to each other, obtained using the Mauve program [15].

The main finding of the work is the detection and mapping of genes responsible for the basic probiotic properties of *L. helveticus* D75 and D76. These genes include those mediating the synthesis of exopolysaccharides, adhesion, utilisation of casein, utilisation of lactose and galactose, genes responsible for the immunomodulatory properties of the bacteria (synthesis of teichoic acids and exopolysaccharides), genes for the synthesis of bacteriocins, genes related to antibiotic resistance, and genes encoding components of the CRISPR-Cas system.

The presence of genes related to the synthesis of exopolysaccharides endows strains with the ability to form a polysaccharide capsule that protects bacteria from environmental damage. Extracellular polysaccharides stimulate the immune system of the host, participate in cell adhesion, improve the rheological properties and texture of fermented foods, and counteract the syneresis of fermented milk [53].

The cell structures responsible for adhesion contribute to the colonisation of the surface of the mucin layer of the gastrointestinal tract, and the subsequent formation of a stable biofilm [54]. Biofilms protect bacteria from host immune factors and antagonistic factors of other members of the microbiota, which ensures close contact of bacteria with each other and with host cells. This latter increases the efficiency of the exchange of biologically active substances and regulatory molecules between bacteria, and between bacteria and host cells.

The presence of a significant number of *mucBP* genes in the genomes of *L. helveticus* D75 and D76 indicates that these strains are adapted for growth on the surface of mucin. Both strains possess fully intact *fpbA* and *fpbIII* fibrinogen adhesion genes. The presence of fibronectin allows bacteria to attach to the surface of gastrointestinal cells through fibrinogen on the outer surface.

The ability to consume milk proteins is a fundamental property of probiotic LAB. By breaking down proteins using extracellular enzymes, lactobacilli acquire substrates for their own nutrition, and biologically active peptides that have a positive effect on the host may be released, such as *Ile-Pro-Pro* and *Val-Pro-Pro* that exert antihypertensive action [55] and prevent inflammatory changes in adipocytes, and, thereby, provide host protection against metabolic diseases [56]. Analysis of the genomes of *L. helveticus* D75 and D76 identified 38 genes encoding proteolytic enzymes and protein transport systems, which indicates the adaptation of these strains to growth on protein-rich substrates such as milk. These include lactocepin H3 (*prtH3*) and lactocepin H (*prtH*), which are key enzymes in the utilisation of milk casein. Lactocepin H3 consist of four domains: S8 peptidase domain (Pfam No. PF00082), a type III fibronectin-like domain (Pfam No. PF06280), and two copies of the surface later A protein domain (Pfam No. PF03217). Lactocepin H consists of an I9 peptidase inhibitor domain (Pfam No. PF05922), an S8 peptidase domain (Pfam No. PF00082), a PA domain (Pfam No. PF02225), a type III fibronectin-like domain (Pfam No. PF06280), and an SlpA domain (Pfam No. PF03217). Based on this structure, it appears that part of the proteinase molecule is immersed in the cell membrane and murein, and its active catalytic domain (S8) protrudes outward into the extracellular space where it interacts directly with casein molecules. In at least some bacterial strains, lactocepin H3 (*prtH3*) is involved in the formation of the biologically active tripeptides *Ile-Pro-Pro* and *Val-Pro-Pro* mentioned above.

As with other lactobacilli, lactose is the main energy substrate for *L. helveticus* D75 and D76. The consumption of lactose by microbiota bacteria leads to a decrease in its concentration, which helps protect people with lactose deficiency from its harmful effects. The presence of the Leloire metabolic pathway in *L. helveticus* D75 and D76 allows these organisms to utilise the galactose, which results from lactose cleavage.

The presence of an I-B type CRISPR system containing 33 spacer sites indicates that, during their evolutionary development, *L. helveticus* D75 and D76 were in close contact with many bacteriophages and foreign genetic elements and, therefore, acquired immunity against them. The CRISPR-Cas type I-B system belongs to a very common class of CRISPR systems. Its main function is to provide adaptive immunity, but it may also be involved in certain genomic rearrangements. The target for this type of adaptive immunity system is double-stranded molecules of foreign DNA. Their recognition occurs through protospacer sequences containing specific protospacer adjacent motifs (PAM). Cas3 proteins, which form the core of the Cascade multi-protein complex, are particularly important [57,58].

One of the most important characteristics of probiotic bacteria is their ability to inhibit the growth of pathogenic bacteria using evolutionarily developed mechanisms of a specific and nonspecific antagonism. Nonspecific antagonism is associated with the constitutive synthesis of metabolites that inhibit the growth of foreign microflora (for example, lactic acid). Specific antagonism is generally a quorum-dependent process during which bacteria produce specific antibacterial substances in response to the presence of foreign microorganisms in the medium. Bacteriocins, which are short polypeptides that form an uncontrolled pore in the target cell’s cell membrane, are the most widely studied molecules related to this process [59].

Genetic analysis indicated that *L. helveticus* D75 and D76 are capable of producing the bacteriocin helveticin J, which belongs to the third class of bacteriocins and destroys the membrane potential of target cells. Currently, the genes involved in the synthesis of bacteriocin helveticin J are not well known. Furthermore, the mechanisms by which the bacteriocin molecule is processed are poorly understood, as are mechanisms that protect bacteria against bacteriocin produced in their own cells. For the production of helveticin J, at least two ORFs are required in which one is related to prebacteriocin. The genetics and mechanisms of the second ORF are poorly understood, but it is assumed that it encodes a surface protein. In some cases, the related genetic cluster may include two additional genes with unknown function [59].

The genetic clusters found in *L. helveticus* D75 and D76 are flanked on both sides by mobile genetic elements. This indicates that they may form the central structure of the transposon. The *helJ* genes in *L. helveticus* D75 and D76 do not share high sequence identity among each other. Apparently, these clusters may be acquired by bacteria through sequential horizontal transfer from different organisms, and this happened before the evolutionary divergence of strains D75 and D76.

The presence of the *EntA_Imm* gene suggests that *L. helveticus* D75 and D76 are resistant to the action of bacteriocins such as enterocins. The presence of this immunity system indicates that the strains evolved in the gastrointestinal tract together with *Enterococcus* bacteria that produce enterocins.

The presence of bile salt hydrolase genes indicates the theoretical resistance of *L. helveticus* D75 and D76 to bile acids. Both strains could grow on MRS medium in the presence of 0.5% concentration of bile salts, which is consistent with published sources indicating a certain degree of resistance to bile salts in dairy bacteria species such as *L. helveticus* and *L. delbrueckii* [60].

In accordance with the European Food Safety Authority (EFSA) requirements for the safety of probiotics, bacterial strains that can potentially be used as probiotics must be characterised in terms of their resistance to clinically important antibiotics. Specifically, antibiotic resistance genes should not be localised on mobile elements such as plasmids or transposons. We could not find a detailed description of strains D75 and D76 antibiotic resistance. However, the authors of the strains [1] noted the presence of their resistance to gentamicin, kanamycin, amikacin, sisomycin, monomycin, tobramycin, and netilmicin. According to the results of this work, antibiotics resistance is likely related to the activity of genes *had*, *vanZ*, *blaZ*, *pbpX*, and *mprF*.

## 5. Conclusions

The genomes of *L. helveticus* D75 and D76, 2,053,066 bp (CP020029 in GenBank) and 2,058,319 bp (CP16827 in GenBank), respectively, were completely sequenced de novo and annotated. Genomic analysis revealed a GC content of 37% for both genomes. The number of pseudogenes formally detected during automatic annotation was 317 and 265 for strains D75 and D76, respectively. ANI tests revealed 99.22% (76.3% overlapping nucleotide sequences) identity between the genomes of D75 and D76, and typical genomes of *L. helveticus* species. Thus, D75 and D76, previously classified as *L. acidophilus* species, were reclassified as *L. helveticus*.

In the genomes of *L. helveticus* D75 and D76, seven key gene groups were identified including genes responsible for the synthesis of exopolysaccharides, genes related to adhesion, casein recycling, utilisation of milk sugars, CRISPR-Cas adaptive immunity, specific bacterial antagonism, and resistance to antibiotic substances. The helveticin J gene is expressed during the growth of both strains.

The identified genes provide *L. helveticus* D75 and D76 with additional benefits and probiotic potential. For example, the protein product of the *prtH3* gene can promote the metabolism of casein by *L. helveticus* D75 and D76 and generate antihypertensive tripeptides. The products of the *mucBP*, *fbpA*, and *fbpIII* genes endow *L. helveticus* D75 and D76 with adhesive properties with respect to the mucin layer and epithelial cells of the human gastrointestinal tract. The presence of adhesins suggests that *L. helveticus* D75 and D76 are able to form biofilms on the surface of the gastrointestinal epithelium, which contributes to the displacement of pathogenic bacteria and more efficient metabolite exchange with the host. The CRISPR-Cas type I-B system provides *L. helveticus* D75 and D76 strains with additional resistance to bacteriophages. The presence of 33 spacers in the CRISPR system of these strains likely provides an opportunity to counteract a wide range of foreign mobile genetic elements.

*L. helveticus* D75 and D76 do not contain mobile genetic elements harbouring genetic determinants of resistance to clinically significant drugs. All identified antibiotic resistance genes are encoded on genomic DNA and are not present of transposons. This indicates the safety of the strains and the extremely low probability of transmission of antibiotic resistance to pathogenic and conditionally pathogenic microorganisms. The *had* gene, detected in the genomes of both *L. helveticus* D75 and D76, presumably allows the strains to withstand the effects of aminobenzoate xenobiotics such as gentamicin, kanamycin, amikacin, sisomycin, monomycin, tobramycin, and netilmicin. The presence of the *blaZ* gene provides *L. helveticus* D75 and D76 protection against beta-lactam antibiotics, and the *vanZ* gene facilitates resistance to low concentrations of the glycopeptide antibiotic teicoplanin. The identified antibiotic resistance genes are common among species in the *Lactobacillus* genus [61].

*L. helveticus* D75 and D76 comply with the safety and efficacy requirements for probiotic preparations stated by the EFSA and U.S. Food and Drug Administration (FDA). They are members of the generally recognized as a safe (GRAS) group, their genomes are fully sequenced, there is no risk of horizontal gene transfer of antibiotic resistance, they possess antagonistic activity against pathogenic and opportunistic bacteria, they exhibit probiotic properties, and they lack genes related to pathogenicity and the synthesis of bacterial toxins.

## Figures and Tables

**Table 1:** Primers used to amplify the *gyrB* housekeeping gene and helveticin J of *L. helveticus* D75 and D76.

Gene Name	The Forward Primer (5′–3′)	The Reverse Primer (5′–3′)	Optimum Primer Annealing Temperature (°C)	Amplified Fragment Length (bp)	Optimum Elongation Temperature (°C)
*gyrB*	GTCCATTTCACCTAACCCCTT	ACCTCTGTACCAAGTCCGTCA	57.2/60.1	136	53.7
*helJ4*	GGCATTTCTTCATCTGGAGC	GGGATGATGGTTCAGGTCAC	56.8/58.3	166	52.5

**Table 2:** Similarity between the nucleotide sequences of genes *helJ1*, *helJ2*, *helJ3*, *helJ4*, and *helJ5* in *L. helveticus* D75 and D76.

D75 D76	helJ1	helJ2	helJ3	helJ4	helJ5
**helJ1**	100%	*	*	*	*
**helJ2**	*	100%	*	*	*
**helJ3**	*	*	100%	*	77%
**helJ4**	*	*	*	100%	*
**helJ5**	*	*	77%	*	100%

* indicates—no similarity found.

**Table 3:** Similarity between the amino acid sequences of the *helJ1*, *helJ2*, *helJ3*, *helJ4*, and *helJ5* proteins in *L. helveticus* D75 and D76.

D75 D76	helJ1	helJ2	helJ3	helJ4	helJ5
**helJ1**	100%	*	59%	51%	65%
**helJ2**	*	100%	*	20%<	*
**helJ3**	59%	*	100%	58%	85%
**helJ4**	51%	20%<	58%	100%	55%
**helJ5**	65%	*	85%	55%	100%

* indicates—no similarity found.

## Supplementary Materials

The following are available online at https://www.mdpi.com/2076-2607/8/3/329/s1. Table S1: Genes of *L. helveticus* D75 and D76 that are encoding proteins involved in the biosynthesis of exopolysaccharide components. Table S2: Genes of *L. helveticus* D75 and D76 are encoding surface adhesive proteins. Table S3: Genes of *L. helveticus* D75 and D76 are encoding proteins involved in casein utilization. Table S4: Genes of *L. helveticus* D75 and D76 are encoding proteins involved in utilization of milk sugars. Table S5: Genes of *L. helveticus* D75 and D76 are encoding proteins involved in the Leloir metabolic pathway. Table S6: Genes of *L. helveticus* D75 and D76 are encoding the CRISPR-Cas system. Table S7: Genes of *L. helveticus* D75 and D76 are encoding proteins involved into specific antibacterial antagonism. Table S8: Genes of *L. helveticus* D75 and D76 are encoding proteins involved in antibiotics and xenobiotics tolerance.
