# Supplementary material for: Whole-Genome Sequencing of Lactobacillus helveticus D75 and D76 Confirms Safety and Probiotic Potential
Source: Microorganisms. 2020 Feb 26;8(3):329. doi: 10.3390/microorganisms8030329 (PMC7142726; doi:10.3390/microorganisms8030329)
Supplement: Supplementary file 1 [file microorganisms-08-00329-s001.pdf]

microorganisms

Table S1. Genes of *L. helveticus* D75 and D76 that are encoding proteins, involved in biosynthesis of exopolysaccharide components.

Gene	Position in the genome of *L. helveticus* D75	Position in the genome of *L. helveticus* D76	Gene translation product
dltB	52716..53954	1485896..1487134	D-alanyl transfer protein involved in the incorporation of D-alanine into lipoteichoic acids
dltD	54245..55531	1487425..1488711	membrane protein involved in the biosynthesis of D-alanyl-lipoteichoic acid
lysA	60027..60964	1493207..1494144	glycoside hydrolase family 25 with lysozyme activity, involved in peptidoglycan catabolic process
eps	61238..62854	1494418..1496034	polysaccharide transporter
hyd	98080..98562	1532643..1533125	hydrolase associated with cell wall catabolism and containing the N1pC / P60 protein domain. This protein carries out the hydrolysis of peptidoglycan during bacterial growth, development and cell division
wzx	299076..300539	1733641..1735104	Flipase transporter, involved in synthesis of exopolysaccharides by polymerization and export
tagA	418185..418913	1852750..1853478	glycosyltransferase of the WecB / TagA / CpsF family. The TagA protein family is involved into biosynthesis of cell wall polyglycerol phosphate
epsE	418921..420021	1853486..1854586	glycosyltransferase with glycosyltransferase family 4 and glycosyltransferase group 1 domains. This protein involved in exopolysaccharides biosynthesis pathway
tagF	426790..427944	1861355..1862509	CDP-glycerol: polyglycerophosphotransferase. This protein participates in the biosynthesis of teichoic acid
epsU	427949..429379	1862514..1863944	capsular biosynthesis protein
gtrA	540636..541175	1975201..1975740	dolichyl-phosphate beta-D-mannosyltransferase is an intergar membrane protein, involved into biosynthesis of polysaccharides
murE	686618..687970	64244..65596	UDP-N-acetylmuramoylalanyl-D-glutamate-2,6-diamino-pimelate ligase. This protein involved into the second step of peptidoglycan biosynthesis
gtrA	744428..744844	122054..122470	dolichyl-phosphate beta-D-mannosyltransferase, an intergar membrane protein involved into biosynthesis of polysaccharides
pbp	760107..761921	137733..139880 547	penicillin-binding protein. This protein involved into the final step of peptidoglycan biosynthesis and encoded by pseudogene
murG	764620..765726	142246..143352	UDP-N-acetylglucosamine--N-acetylmuramyl-(pentapeptide) pyrophosphoryl-undecaprenol N-

			acetylglucosamine transferase. This peptide involved into the peptidoglycan biosynthesis pathway
pbp	980532..981629	358127..359224	penicillin-binding protein. Involved into the final step of peptidoglycan biosynthesis.
ykuD	983163..984368	360758..361963	transpeptidase with peptidoglycan binding domain. This protein gives rise to an alternative pathway for peptidoglycan cross-linking
pbp1A	1158878..1161178	536232..538532	Membrane penicilin-binding carboxypeptidase 1A. This protein involved into the final step of peptidoglycan biosynthesis.
lys	1329788..1331023	708522..709757	protein with lysozyme activity and surface later protein A domain. This protein involved into peptidoglycan catabolic process
pbp2B	1450192..1452300	828926..831034	membrane penicilin-binding carboxypeptidase 2B with transpeptidase domain. This protein involved into the final step of peptidoglycan biosynthesis.
ykuD	1576492..1577166	956606..957280	transpeptidase with YkuD domain. It has been shown that domain YkuD can act as an L,D-transpeptidase that gives rise to an alternative pathway for peptidoglycan cross-linking
dacA	1578143..1579438	958257..959552	D-alanyl-D-alanine carboxypeptidase. This protein involved into the peptidoglycan biosynthesis and degradation process

Table S2. Genes of *L. helveticus* D75 and D76 that are encoding surface adhesive proteins.

Gene	Position in the genome of *L. helveticus* D75	Position in the genome of *L. helveticus* D76	Gene translation product
mucBP	383312..383779	1817877..1818344	mucin binding protein, encoding by hypothetical gene
mucBP	873784..874104	251411..251731	mucin binding protein
mucBP	874101..874787	251728..252414	mucin binding protein
mucBP	874774..875076	252401..252703	mucin binding protein, encoding by hypothetical gene
mucBP	875124..875291	252751..252918	mucin binding protein
fbpA	1144883..1146577	522237..523931	fibronectin
slpA	1219708..1220208	597062..597562	S-layer protein
srtA	1226873..1227562	604227..604916	class A sortase
mucBP	1584744..1585364	964858..965478	mucin binding protein, encoding by hypothetical gene
slpA	1823168..1823740	1203282..1203854	S-layer protein
fbpIII	1847693..1849087	1227807..1229201	protein with fibronectin type III domain
slpA	1866469..1867833	1246583..1247947	S-layer protein
slpA	1989578..1990480	1369692..1370594	S-layer protein

Table S3. Genes of *L. helveticus* D75 and D76 that are encoding proteins, involved in casein utilization.

Gene	Position in the genome of *L. helveticus* D75	Position in the genome of *L. helveticus* D76	Gene translation product
pepN	208194..210728	1642757..1645291	intracellular peptidase
dtpT	210838..211719	1645401..1646267	proton-dependent oligopeptide transporter
pepD4	217882..219294	1652447..1653859	D4 family dipeptidase
yvpB	264253..264966	1698818..1699531	cysteine protease
pepA	478203..479285	1912768..1913850	A family intracellular peptidase
pepM24	541758..542585	1976323..1977150	M24 family cobalt-dependent methionine aminopeptidase
pepM16	590270..591484	2026215..2027429	M16 family peptidase
clpP	622701..623285	327..911	ATP-dependent protease
pepCE	882452..883765	260079..261392	CE family cysteine peptidase
pepT2	985911..987197	363506..364792	T2 family intracellular tripeptidase
pepM20	1050472..1051737	428066..429331	M20 family peptidase
prtH	1059720..1065005	437314..440053	extracellular proteinase lactocepin H (in *L. helveticus* D76 encoded by pseudoene)
pepO2	1066263..1068209	443550..445496	O2 family endopeptidase
pepV	1088319..1089722	465606..467009	intracellular dipeptidase
pepT1	1190204..1191445	567558..568799	T1 family intracellular tripeptidase
pepM23	1205766..1206221	583120..583575	M23 family peptidase
pepO	1257415..1259358	634769..636712	O family endopeptidase
pepD2	1278053..1279474	656787..658208	D2 family dipeptidase
oppA	1285191..1286711	663925..665445	periplasmic oligopeptide-binding protein with transmembrane transporter activity
oppA	1287156..1288907	665890..667641	periplasmic oligopeptide-binding protein with transmembrane transporter activity
oppC	1289113..1290042	667847..668776	periplasmic oligopeptide transport protein
oppB	1290057..1291016	668791..669750	periplasmic oligopeptide transport protein
oppF	1291019..1292005	669753..670739	ATP-binding oligopeptide ABC transporter
oppD	1292009..1293043	670743..671777	ATP-binding oligopeptide ABC transporter
pepQ2	1319996..1321105	698730..699839	Q2 family Pro-Xaa proline peptidase
pepX	1333459..1335840	712193..714574	intracellular x-prolyl-dipeptidyl peptidase
pepQ	1382902..1383483	761636..762217	intracellular peptidase
pepQ	1383486..1384103	762220..762837	intracellular peptidase
oppA3	1384117..1385724	762851..764458	periplasmic oligopeptide-binding protein with transmembrane transporter activity, encoded by pseudogene
pepM1	1508274..1509788	887008..888522	M1 family membrane alanine peptidase
prtH3	1516632..1521602	895366..900336	extracellular proteinase lactocepin H3
prtP	1588098..1589006	968212..969120	putative cell wall associated proteinase
pepQ	1632307..1633413	1012421..1013527	Pro-Xaa peptidase
pepC	1716220..1717569	1096333..1097682	C family peptidase
pepD	1808406..1809830	1188520..1189944	D family dipeptidase
pepG	1843780..1845093	1223894..1225207	E2 family intracellular peptidase
pep	1907056..1908292	1287170..1288406	serine protease
pepI	1940701..1941585	1320815..1321699	I family Pro-Xaa peptidase

Table S4. Genes of *L. helveticus* D75 and D76 that are encoding proteins, involved in utilization of milk sugars.

Gene	Position in the genome of *L. helveticus* D75	Position in the genome of *L. helveticus* D76	Gene translation product
glcU	153033..153920	1587596..1588483	glucose transporter
lctP2	253742..254791	1688498..1689356	L-lactate permease, encoded by pseudogene
lacS	1426981..1428897	805715..807631	lactose permease
lacR	1433031..1434038	811765..812772	transcriptional regulator
lacL	1435240..1437126	813974..815860	large subunit of beta-galactosidase
lacM	1437110..1438066	815844..816800	small subunit of beta-galactosidase
ldh	1779366..1780337	1159479..1160450	lactate dehydrogenase

Table S5. Genes of *L. helveticus* D75 and D76 that are encoding proteins, involved in Leloir metabolic pathway.

Gene	Position in the genome of *L. helveticus* D75	Position in the genome of *L. helveticus* D76	Gene translation product
galM	1377923..1378918	756657..757652	galactose mutarotase
galT	1379039..1380502	757773..759236	galactose-1-phosphate uridyltransferase
galK	1380524..1381690	759258..760424	galactokinase
galE	1438171..1439163	816905..817897	UDP-galactose 4-epimerase

Table S6. Genes of *L. helveticus* D75 and D76 that are encoding CRISPR-Cas system.

Gene	Position in the genome of *L. helveticus* D75	Position in the genome of *L. helveticus* D76	Gene translation product
CRISPR1	1360958..1361168	739692..739902	first CRISPR array
CRISPR2	1490768..1491325	869502..870059	second CRISPR array
CRISPR3	1492138..1493550	870872..872284	third CRISPR array
cas2	1493735..1494016	872469..872750	type I-B CRISPR-associated endonuclease Cas2
cas1	1494022..1495011	872756..873745	type I-B CRISPR-associated endonuclease Cas1
cas4	1495021..1495512	873755..874246	type I-B CRISPR-associated endonuclease Cas4
cas3	1495528..1497957	874262..876691	type I-B CRISPR-associated helicase / Cas3 endonuclease
cas5	1498100..1498813	876834..877547	type I-B CRISPR-associated protein Cas5
cas7	1498800..1499702	877534..878436	type I-B CRISPR-associated protein Cas7
cas8b1	1499721..1501478	878455..880212	type I-B CRISPR-associated protein Cas8b1
cas6	1501496..1502251	880230..880985	type I-B CRISPR-associated endoribonuclease Cas6

Table S7. Genes of *L. helveticus* D75 and D76 that are encoding proteins, involved into specific antibacterial antagonism.

**Gene**	**Position in the genome of *L. helveticus* D75**	**Position in the genome of *L. helveticus* D76**	**Gene translation product**
slpA1	196015..197187	1630578..1631750	surface layer protein, probably involved in bacteriocin biosynthesis
helJ1	197306..198268	1631869..1632831	class III bacteriocin helveticin J. Peptide toxin produced by bacteria to inhibit the growth of a similar or closely related bacterial strain
helJ2	198298..198612	1632861..1633175	open reading frame of bacteriocin class III helveticin J
cvpA	225735..226271	1660300..1660836	CvpA family protein. This protein participates in the synthesis of colicin V bacteriocin (*cvaC* gene)
entI	241697..242020	1676262..1676585	enterocin A immunity protein
entI	255040..255312	1689605..1689877	enterocin A immunity protein, encoded by hypothetical gene
helJ3	467742..468227	1902307..1902792	class III bacteriocin helveticin J. Peptide toxin produced by bacteria to inhibit the growth of a similar or closely related bacterial strain. This protein encoded by hypothetical gene
slpA2	468321..469397	1902886..1903962	surface layer protein, probably involved in bacteriocin biosynthesis
entI	518263..518628	1952828..1953193	enterocin A immunity protein
slpA3	1509877..1510854	888611..889588	surface layer protein, probably involved in bacteriocin biosynthesis
helJ4	1507159..1508136	885893..886870	class III bacteriocin helveticin J. Peptide toxin produced by bacteria to inhibit the growth of a similar or closely related bacterial strain.
slpA4	1789582..1790658	1169695..1170771	surface layer protein, probably involved in bacteriocin biosynthesis
helJ5	1790748..1791497	1170861..1171610	class III bacteriocin helveticin J. Peptide toxin produced by bacteria to inhibit the growth of a similar or closely related bacterial strain.

Table S8. Genes of *L. helveticus* D75 and D76 that are encoding proteins, involved into antibiotics and xenobiotics tolerance.

Gene	Position in the genome of *L. helveticus* D75	Position in the genome of *L. helveticus* D76	Gene translation product
vanZ	664276..664791	41902..42417	membrane protein that confers a low level of resistance to the glycopeptide antibiotic teicoplanin
vanZ	681652..682266	59278..59892	membrane protein that confers a low level of resistance to the teicoplanin antibiotic
blaZ	685285..686298	62911..63924	class C beta-lactamase protein that providing multi-resistance to β-lactam antibiotics
pbpX	818620..819702	196246..197328	penicillin binding protein containing beta-lactamase domain
mprF	1610666..1611692	990780..991806	Phosphatidylglycerol lysyltransferase, encoded by pseudogene
had	1626502..1627281	1006616..1007395	4-nitrophenylphosphatase. This protein carries out biodegradation of aminobenzoate xenobiotics by acting on ester bonds
terC	1922220..1923008	1302334..1303122	tellurium resistance protein embedded in the bacterial cell membrane
pbpX	1977115..1978125	1357229..1358239	penicillin binding protein containing beta-lactamase domain
